# Exploring miR-21 Knock-Out Using CRISPR/Cas as a Treatment for Lung Cancer

**DOI:** 10.3390/genes16020133

**Published:** 2025-01-24

**Authors:** Patricia Lara, Araceli Aguilar-González, Francisco Martín, Cristina Mesas, Javier Moreno, Ana R. Rama

**Affiliations:** 1 Institute of Biopathology and Regenerative Medicine (IBIMER), Center of Biomedical Research (CIBM), University of Granada, 18100 Granada, Spain; 2 GENYO, Centre for Genomics and Oncological Research: Pfizer, University of Granada, Andalusian Regional Government PTS Granada, 18016 Granada, Spain; 3 Department of Medicinal & Organic Chemistry and Excellence Research Unit of “Chemistry Applied to Biomedicine and the Environment”, Faculty of Pharmacy, University of Granada, Campus de Cartuja, 18071 Granada, Spain; 4 Instituto de Investigación Biosanitaria de Granada, ibs. GRANADA, 18014 Granada, Spain; 5 Departamento de Bioquímica y Biología Molecular e Inmunología, Facultad de Medicina, Universidad de Granada, 18071 Granada, Spain; 6 Department of Anatomy and Embryology, Faculty of Medicine, University of Granada, 18016 Granada, Spain; 7 Department of Health Sciences, University of Jaén, 23071 Jaén, Spain

**Keywords:** miRNA, miR-21, CRISPR/Cas9, lung cancer, A549 cells

## Abstract

Background: Lung cancer is a leading cause of cancer-related deaths worldwide. Its high incidence and poor prognosis demonstrate the need to investigate new therapies. The PI3K/AKT pathway is activated in carcinogenic processes such as invasion, proliferation, and drug resistance. MiR-21 is a microRNA overexpressed in numerous types of cancer and which activates PI3K/AKT pathway by down-regulating its main targets, *PTEN* and *PDCD4*. CRISPR is a revolutionary gene-editing technology that allows genes to be deleted. The aim of this study was to use CRISPR/Cas9 technology as an option to reduce carcinogenic and drug resistance processes by eliminating miR-21. Methods: CRISPR/Cas9 was used to knock out miR-21 (miR-21 KO) in A549 lung cancer cells and thus reverse the carcinogenic processes activated by miR-21 overexpression. Furthermore, the effect of miR-21 KO on drug resistance was studied, choosing the main chemotherapeutic agents used for the treatment of lung cancer: gemcitabine, carboplatin, paclitaxel, and oxaliplatin. Results: miR-21 KO A549 cells exhibited a reduction in proliferation, migration, and colony formation compared to A549 cells. In contrast, the expression of *PTEN* and *PDCD4* increased in miR-21 KO A549 cells. Furthermore, miR-21 KO A549 cells showed a decrease in the IC_50_ of the drugs used for the treatment of lung cancer: gemcitabine, carboplatin, paclitaxel, and oxaliplatin. Conclusions: Based on these results, miR-21 knock-out using CRISPR/Cas could be a promising strategy for the treatment of lung cancer.

## 1. Introduction

Lung cancer is the second of the most common and deadly types of cancer worldwide. It is considered the leading cause of cancer-related mortality in both women and men [1]. The 5-year survival rate from the diagnosis of the disease is much lower (17.4%) than those of other types of cancer, mainly due to its late diagnosis in advanced stages [2]. Specifically, 2.2 million new cases of lung cancer (11.4%) and almost 1.8 million deaths from lung cancer (18.0%) were estimated in 2020 [3]. According to histopathology, two types of lung cancer are distinguishable, non-small cell lung cancer (NSCLC) (85%) and small-cell lung cancer [4]. Chemotherapy is the main treatment, although its effectiveness may be limited by the development of drug resistance. Lung cancer’s high incidence, late detection, poor prognosis, and relapses, along with the ineffectiveness of chemotherapy, demonstrate the need to research new therapies to address this disease.

Currently, numerous studies confirm the importance of abnormal miRNA expression in tumor cells. miRNAs are frequently overexpressed in certain types of cancer (miR-21, miR-221/222 cluster, miR-17-92) and decreased in others (miR-15, miR-34, miR-16), triggering an increase in the expression of cancer cell markers and an increase in tumorigenicity [5]. This unusual expression has inspired the development and research of new antitumor therapies, highlighting the use of microRNA (miRNA) as a therapeutic target [6]. Recent advances, such as the targeted administration of miR-34a, have demonstrated the potential for a reduction in the proliferation and tumorigenicity of breast, prostate, and lung cancer [7,8], or an increase in apoptosis and reduction in thyroid cancer proliferation by inhibiting miR-17-5p [9].

miRNA21 (miR-21) is located on chromosome 17 (17q.23.1) in intron 11 of TMEM 49 (transmembrane protein 49), precursor of VMP1 (vacuole membrane protein 1) [10]. The overexpression of miR-21 has been detected in numerous types of cancers, such as lung, breast, stomach, or colon, among others, which is why it is considered a powerful molecular biomarker [11] for the diagnosis, prediction, and prognosis of the disease [12]. Overexpression of miR-21 has been linked to various carcinogenic mechanisms, such as cell proliferation, inhibition of apoptosis or migration, and chemotherapy resistance [13]. Chemotherapy remains the main treatment for lung cancer, with gemcitabine (Gem), carboplatin (Car), paclitaxel (Pac), and oxaliplatin (Oxa) being the main chemotherapeutic agents used. Gem is a standard chemotherapeutic agent for the treatment of lung cancer [14]. Car and Oxa have emerged as alternatives to cisplatin, also used in the first-line treatment of NSCLC, to reduce its side effects [15,16]. On the other hand, Pac, an antimicrotubule agent, is considered one of the most commonly used agents in combination with platinum agents for the treatment of NSCLC [17]. Although chemotherapy remains the main treatment option for lung cancer patients, the emergence of resistance to chemotherapeutic agents poses a major challenge to patient survival [18].

The main targets of miR-21 are programmed cell death protein 4 (*PDCD4*) and the phosphatase and tensin homolog (*PTEN*) [19]. *PDCD4* is a suppressor of tumor progression that leads to cell cycle arrest, increased apoptosis, and increased sensitivity of tumor cells to drugs [20]. However, its expression is down-regulated in several types of cancer such as hepatocellular carcinoma, breast cancer, ovarian cancer, and lung cancer [21,22]. *PTEN*, the other tumor suppressor protein, acts by inhibiting the PI3K/AKT pathway, which is responsible for regulating carcinogenic processes such as invasion, proliferation, and resistance to chemotherapeutic drugs [23]. Overexpression of miR-21 causes tumorigenesis by negatively regulating *PTEN* and *PDCD4* [24].

Based on the above data, new tools are needed for the treatment of lung cancer. Genome-editing tools have emerged as the new option for disease modeling, diagnosis, and therapeutics, which work by achieving precise genome modifications on their DNA or RNA targets [25,26]. In particular, the CRISPR/Cas9 system [27] revolutionized the genome-editing applications due to its versatility, reduced cost, and the possibility of multiplexing [28]. The possibilities for gene therapy applications are immense, from gene deletion [29,30] and gene correction to specific gene insertions into the desired locus [31,32]. However, due to delivery issues with the other applications, the generation of indels for gene knock-out is the first application to have been investigated in clinical trials for the treatment of cancer, infectious diseases, and metabolic disorders [33].

One of the main goals of CRISPR/Cas9 for cancer treatment is to find genotype-specific lesions. Targeted deletion of these damaged genes can decrease the viability of tumor cells, which is a strategy for finding potential targets for cancer therapy [34]. In tumor cells, oncogenes are usually mutated or overexpressed, preventing cell death and, therefore, continuing cell survival and proliferation. In this context, CRISPR technology has been used to knock out oncogenes. An example is the deletion of interleukin 30 (IL)-30, which correlates with progression and high-grade disease, in pancreatic and colorectal cancer cells to prevent disease progression [35,36]. The use of exosomes with plasmid vectors encoded by CRISPR/Cas9 for the elimination of the oncogenic KrasG12D allele in pancreatic cancer cells has achieved the inhibition of proliferation and suppression of tumor growth [37]. Myc is another well-known oncogene, a transcription factor responsible for coordinating the expression of intra- and extracellular functions necessary for cell growth. Myc is overexpressed in several types of cancer, which is why studies have been carried out in lung cancer, using CRISPR technology to suppress the expression of this gene by creating Myc knock-out cells, thereby reducing tumor malignancy [38]. CRISPR/Cas9 has also been used to eliminate miRNAs overexpressed in different cancers. Overexpression of miR-142 was observed in hematopoietic cells and in diffuse large B-cell lymphoma (DLBCL). Deletion of miR-142 using the CRISPR/Cas9 tool decreased tumor malignancy in DLBCL cells [39]. Likewise, CRISPR/Cas9 has also been used to delete miR-371, miR-372, and miR-373, involved in processes of self-renewal, apoptosis, oncogenesis, and drug resistance, in oral squamous cell cancer (OSCC). The results showed OSCC oncogenicity and sensitivity to drugs such as cisplatin [40].

Therefore, CRISPR/Cas9 technology could be considered an option to reduce carcinogenic and drug resistance processes by eliminating miR-21.

## 2. Materials and Methods

### 2.1. Cell Culture

In this study, we used A549 cells (European Cell Culture Collection), representing lung cancer, and miR-21 KO A549 cells, representing edited lung cancer, where the miR-21 sequence was deleted by CRISPR-Cas9. Both were cultured in DMEM (Dulbecco’s Modified Eagle Medium) with 10% FBS (Fetal Bovine Serum) at 37 °C and in 5% CO_2_.

### 2.2. Generation of miR-21 KO A549 Cells by Cas9 RNP Nucleofection

We used the CRISPOR design tool (CRISPOR.org) to design two gRNAs, gRNA1 (GTCTGATAAGCTACCCGACA) and gRNA2 (ATGTCAGACAGCCCATCGAC). For the formation of RNP, chemically synthesized gRNA obtained from IDT (Coralville, IA, USA) (100 μM) was mixed in a 1:3 ratio by volume with high-fidelity Cas9 (IDT, Coralville, IA, USA) and incubated at room temperature for 15 min to form RNP. For gene disruption, A549 cells (2 × 10^5^) (passage 3) were nucleofected with RNP. Nucleofection was performed with an Amaxa Nucleofector 4-D and solution SF (Lonza, Basel, Switzerland), applying program CD-137 in 20 μL Nucleocuvette™ strips.

### 2.3. ICE (Inference of CRISPR Edits)

Genomic DNA from A549 cells was extracted 5 days after nucleofection using a QIAamp DNA mini kit (Qiagen, Germantown, MD, USA) following the manufacturer’s instructions. The genomic regions flanking the CRISPR target site for each treatment were amplified by PCR using different pairs of primers with KAPA Taq PCR (Kapa Biosystems): F-21: GGGGATTTCTTGGTTTGTGAA and R-21: ATACAGCTAGAAAAGTCCCTGAAAA. The fragments were purified with the QIAquick PCR product purification kit (Qiagen), in accordance with the manufacturer’s protocols. To analyze allele modification frequencies, we used ICE (Inference of CRISPR Edits), a web-based analysis tool developed by Synthego (https://ice.synthego.com/#/) (accessed on 20 June 2022). Our purified PCR products were Sanger sequenced using both primers. Then, each sequence chromatogram was analyzed with the ICE software (0.11.8.1) (Synthego, Silicon Valley, CA, USA). Analyses were performed using a control sequence. The ICE score showed editing by NHEJ.

### 2.4. Proliferation Assay (Sulforhodamine B, SBR)

To perform the experiment, 4 × 10^3^ cells/well of A549 cells and miR-21 KO A549 cells, both in a 48-well NUNC^®^ plate, were seeded in a volume of 350 µL/well. Staining with SBR was performed 24, 48, and 72 h after seeding. The experiment was performed in triplicate.

The cells were fixed by decanting the medium and adding 250 µL of TCA at 4 °C, after which the plates were incubated for 20 min at 4 °C. The plates were then washed 3 times with deionized water and allowed to dry. Once dry, 250 µL of 0.08% SBR was added per well and the plates were incubated at room temperature for 20 min under shaking. After incubation, the stain was removed from the plate, which was washed 3 times with acetic acid and left to dry in the oven. Finally, 250 µL of Tris Base was added to lift the dye, and we proceeded with the measurement of the plates using a spectrophotometer.

Reading of the staining results was carried out using a Multiskan EX microplate reader and Ascent Software 2.2 for Multiskan.

### 2.5. Wound-Healing Assay

We seeded 20 × 10^4^ cells/well of A549 cells and miR-21 KO A549 cells in 12-well NUNC^®^ plates. Once 90% confluence was reached, wounding was performed using a 100 µL pipette tip. Finally, the medium was changed to serum-free medium.

Cell migration was monitored by taking images every 24 h after wounding for both using a Leica microscope (Wetzlar, Germany). The results were analyzed using the MRI Wound Healing Tool of ImageJ Software, version 1.54. The experiment was performed in triplicate. Migration was calculated according to the following formula:

Migration (%) = 100 − ((area of the wound time X/area of the wound time 0) × 100)

### 2.6. Colony Formation

A549 cells and miR-21 KO A549 cells were seeded in 6-well plates (400 cells/well). One week after seeding, the cells were fixed with 70% ethanol and stained with 0.5% crystal violet (1 mL/well) for 15 min. Once dried, the number of colonies was determined using ImageJ Software. The experiment was performed in triplicate.

### 2.7. Determination of Half-Maximal Inhibitory Concentration (IC_50_)

We seeded 4 × 10^3^ cells/well of A549 and miR-21 KO A549 cell lines in 48-well plates. Then, they were exposed to a concentration gradient for each of the drugs: gemcitabine (Gem), carboplatin (Car), paclitaxel (Pac), and oxaliplatin (Oxa). Additionally, a no-drug control group was included in each experiment. After 72 h, cells were stained using the SBR protocol, described above, to determine the IC_50_. Each experiment was performed in triplicate.

### 2.8. RNA Extraction and Quantitative Real-Time PCR

Total extraction of A549 cells and miR-21 KO A549 cells was performed with Trizol Reagent (RNeasy Mini Kit, Qiagen, MD, USA), and cDNA was obtained by MMLV-RT (Promega, Madison, WI, USA) using a retrotranscriptase kit, following the manufacturer’s instructions.

Real-time PCR was performed using SYBR Green Supermix (Taq Universal SYBR Green Supermix) (Bio-rad). The genes to be analyzed were *PTEN* and *PDCD4*; gryceraldehyde-3-phosphate dehydrogenase (*GADPH*) was used as an endogenous control, with which gene expression data were normalized. Expression levels were calculated by applying the 2^−∆∆Ct^ method. All quantitative PCR assays were performed on an ABI 7900 system (ABI). Each experiment was performed in triplicate.

### 2.9. Statistical Analysis

The results obtained were expressed as the mean ± standard deviation (SD). For statistical analysis, GraphPad Prism v9.3 software was used, and an ANOVA test was performed to compare the treatments. Results with a *p*-value < 0.05 were considered significant.

## 3. Results

### 3.1. Generation of A549 miR-21 Knock-Out Cell Models by CRISPR/Cas9

**Figure 1:**
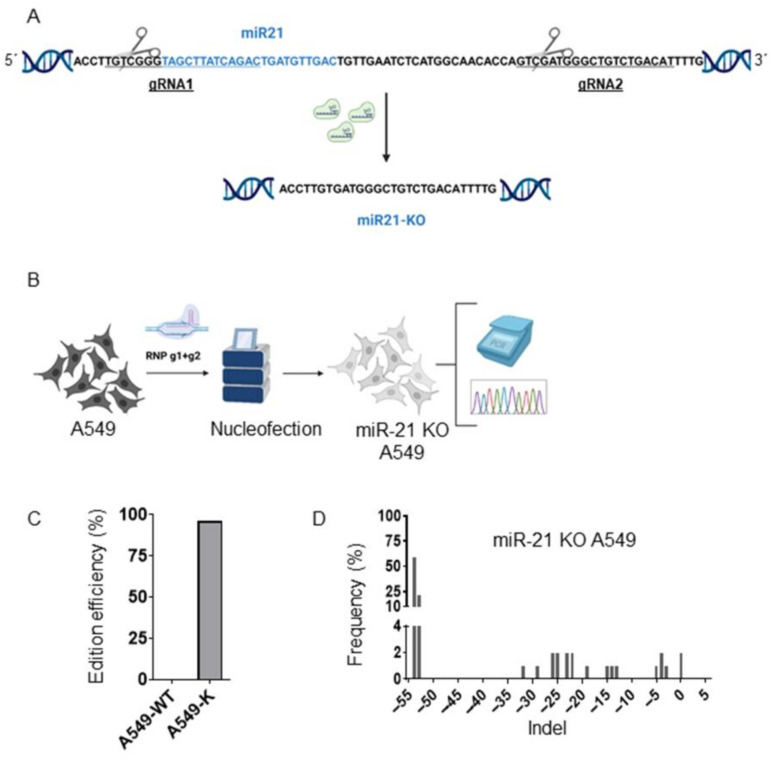
Strategy to knock out miR-21 in A549 cells. (**A**) Schematic of the strategy designed to generate miR-21 KO A549 cells. (**B**). Representative scheme of the nucleofection process with RNP and analysis of the edited cells. Image created with https://www.biorender.com/ (23 September 2023). (**C**) Editing efficiencies of CRISPR/Cas9 targeted in miR-21, determined by the ICE algorithm (see M&M). (**D**) Graph showing the profile of indels generated in miR-21 KO A549 cells nucleofected using the ICE algorithm. The coordinate zero represents the cutite, negative values represent deletions of different lengths, and positive values represent insertions.

miR-21 knock-out (miR-21 KO) in A549 lung cancer cells was performed following the scheme in Figure 1. After the design of gRNA1 and −2 (Figure 1A), both were nucleofected with Cas9 as RNPs (see M&M) into A549 cells to carry out miR-21 knock-out (Figure 1B). We observed around 97% of cells harboring indels (Figure 1C), with a predominant deletion of −54 and −53 nucleotides, corresponding to the deletion of miR-21 (Figure 1D).

### 3.2. MiR-21 Knock-Out Decreases Proliferation, Migration, and Colony Formation

**Figure 2:**
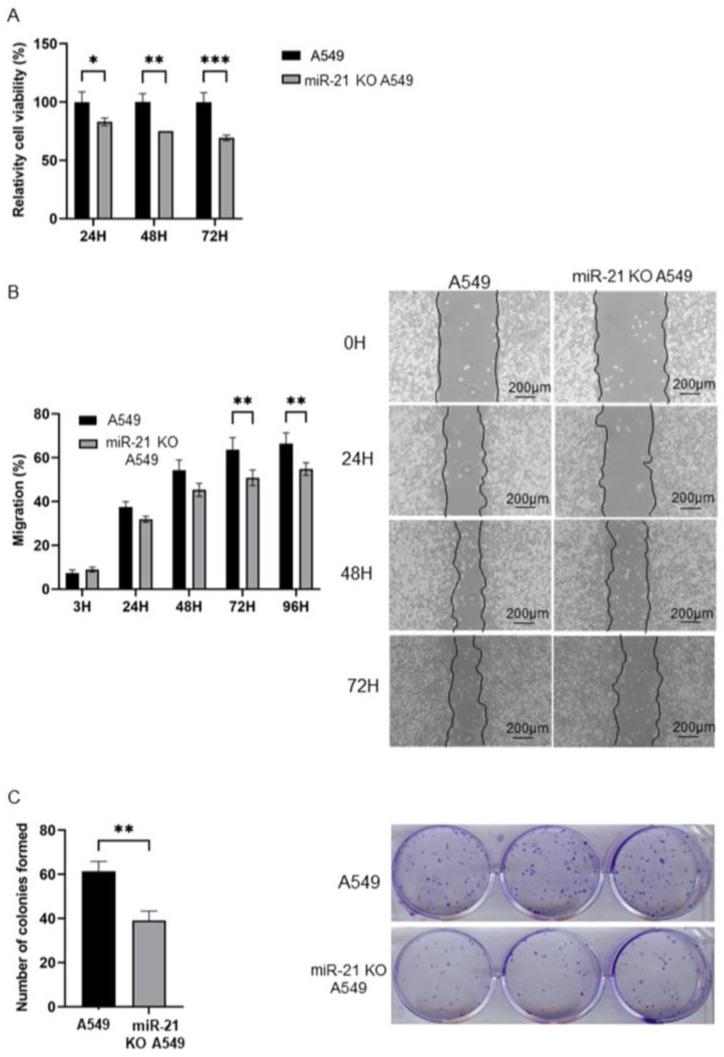
Proliferation, migration, and colony formation analysis of miR-21 KO A549 cells. (**A**) Graphical representation of cell growth of A549 cells and miR-21 KO A549 cells. Statistical analysis performed by ANOVA test. Values represent means ± SD (n = 3). * *p* < 0.1 ** *p* < 0.01, and *** *p* < 0.001. (**B**) Graphical representation of the percentage of cell migration in A549 cells and miR-21 KO A549 cells (area of the scratch). Statistical analysis performed by ANOVA test. Values represent means ± D (n = 3). ** *p* < 0.01. Representative microscopic images (right) of wound-healing assays. Scale bar: 200 µm. (**C**) Graphical representation of the number of colonies formed by A549 cells and miR-21 KO A549 cells. Statistical analysis performed by ANOVA test. Values represent means ± D (n = 3). ** *p* < 0.01. Representative image (right) of colony formation.

Overexpression of miR-21 has been related to carcinogenic processes such as migration, proliferation, and colony formation. We therefore studied the possible variation in these processes after miR-21 knock-out in A549 cells. MiR-21 KO A549 cells showed a time-dependent decrease in cell growth versus A549 cells (overexpression of miR-21), reaching the maximum growth inhibition at 72 h (30.77%) (*p* < 0.001) (Figure 2A). This fact suggested that miR-21 knock-out was related to a marked decrease in cell proliferation.

On the other hand, our wound-healing assay displayed a significant decrease in the cell migration capacity of miR-21 KO A549 cells compared to A549 cells. The greatest inhibition of migration (49.18%) (*p* < 0.01) was observed at 72 h after wounding in miR-21 KO A549 cells (Figure 2B). Finally, the effects of miR-21 KO on colony formation were corroborated in miR-21 KO A549 cells, which exhibited a lower number of colonies (39) than in A549 cells (61) (Figure 2C).

### 3.3. MiR-21 Knock-Out Increases Carboplatin and Paclitaxel Activity

**Figure 3:**
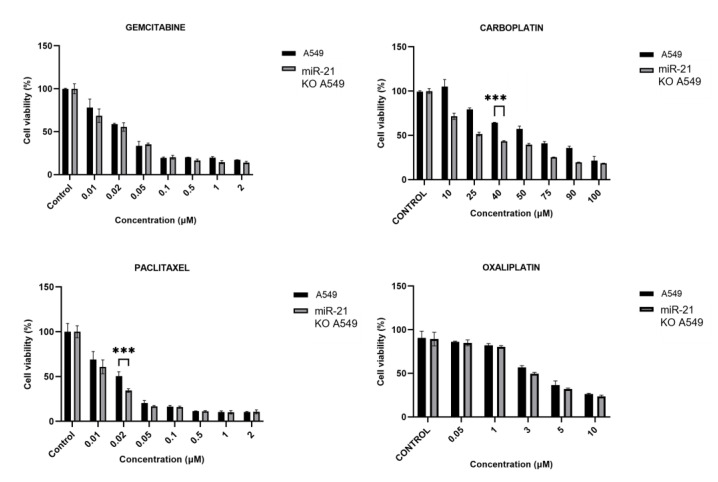
Graphical representation of the IC_50_ results of gemcitabine, carboplatin, paclitaxel, and oxaliplatin. Statistical analysis performed by ANOVA test. Values represent means ± D (n = 3). *** *p* < 0.001.

Gemcitabine (Gem), carboplatin (Car), paclitaxel (Pac), and oxaliplatin (Oxa) were the drugs chosen to study the effect of miR-21 KO on the drug resistance of lung cancer. A decrease in the IC_50_ of Carb (27.65 µM vs. 61.17 µM) and Pac (0.014 µM vs. 0.02 µM) was observed in the miR-21 KO A549 line vs. the A549 cell line (Figure 3).

### 3.4. Gene Expression Analysis

Following the results described above, a gene expression analysis of the main targets of miR-21 (*PTEN* and *PDCD4*) was carried out.

**Figure 4:**
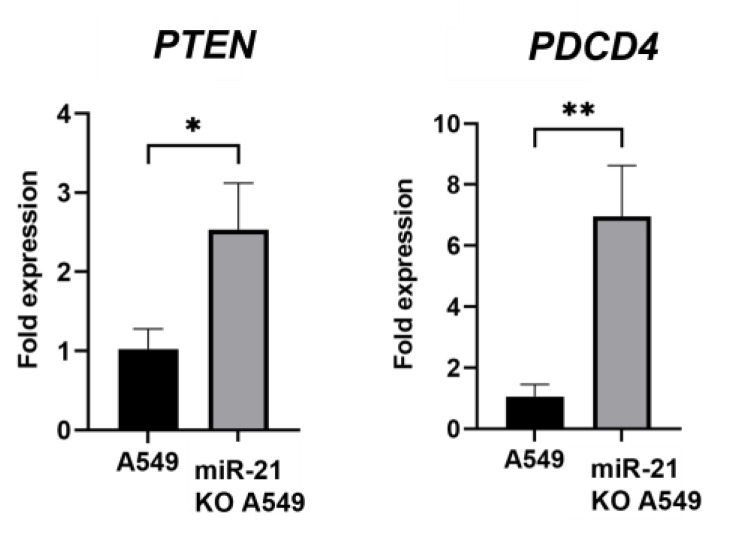
Analysis of *PTEN* and *PDCD4* expression. Relative expression levels were calculated upon *GADPH* (housekeeping gene) normalization, respectively. Statistical analysis performed by ANOVA test. Values represent means ± D (n = 3). * *p* < 0.1 ** *p* < 0.01.

miR-21 KO A549 cells were revealed to have a higher expression of both *PTEN* (1.17-fold; *p* < 0.1) and *PDCD4* (4.22-fold; *p* < 0.01) than in A549 cells (Figure 4).

## 4. Discussion

Lung cancer is considered the leading cause of cancer-related mortality in the world, with non-small-cell lung cancer (NSCLC) being the most prevalent lung cancer, accounting for 85–90% of all lung cancers [41]. Its high incidence and resistance to chemotherapy make it necessary to look for new therapies. miR-21 has been reported as an oncogene, which is overexpressed in several malignant tumors [42], thus bearing the potential to be used as a prognostic or diagnostic marker for tumors [43].

The aim of this study was to test the efficacy of using CRISPR/Cas9 to knock down miR-21, and we chose the A549 cell line as a model of NSCLC cancer line because it has overexpressed miR-21 [44,45]. We used CRISPR/Cas9 to knock out miR-21 (miR-21 KO) in A549 lung cancer cells. miR-21 KO A549 cells showed a time-dependent decrease in proliferation, migration, and colony formation; however, this was not observed in A549 cells. Li H. et al. [2] demonstrated that increased expression of miR-21 following transfection with a mimetic in A549 cells promoted cell proliferation compared to non-transfected A549 cells. On the contrary, a decrease in cell migration was observed in A549 cells transfected with a miR-21 inhibitor versus non-transfected A549 cells [46]. Similarly, A549 cells transfected with a circular sponge against miR-21 showed decreased proliferation, migration, and colony formation compared to non-transfected A549 cells [45]. miR-21 KO by CRISPR/Cas9 has also been used in other types of tumors such as ovarian cancer [47] or glioblastoma, and it has produced results similar to ours. A significant reduction in cell proliferation, migration, and invasion was detected after miR-21 KO in GL261, CT2A mouse glioma, and U87 human glioma, as well as a reduction in tumor growth during in vivo assays [48].

miR-21 has been linked to drug resistance processes, and in our study, we included some of the main drugs used as chemotherapy for the treatment of lung cancer. Our results show that in the A549 cell line, miR-21 KO is more sensitive to carboplatin and paclitaxel than in the basal A549 cell line, suggesting that miR-21 may play a crucial role in mediating resistance to these chemotherapeutic treatments. Liu et al. [49] demonstrated that the inhibition of miR-21 by a small interfering RNA, siRNA, increased the sensitivity of hepatocellular carcinoma cell lines to Car. In contrast, overexpression of miR-21 by mimics increases cell viability, reduces cellular sensitivity to Car, and increases the expression of the ABCB6 efflux pump [50]. Gamal-Eldeen et al. [51] used perfluorodecalin, an oxygen transporter, in A549 to inhibit miR-21 expression. Combined treatment of Car and perfluorodecalin significantly suppressed miR-21 expression, thereby increasing the efficacy of Car. In relation to PTX, Su et al. [52] used the miR-21 inhibitor in combination with PTX in the A549 cell line, demonstrating a significant decrease in cell growth compared to the wild-type A549 cell line. In this regard, the miR-21 inhibitor improved the efficacy of PTX, achieving a 54–63% reduction in cell viability in cervical cancer cells [53]. Similar figures were found in the MCF7 breast cancer cell line, where a miR-21 inhibitor decreased the IC_50_ of PTX [54].

On the other hand, *PTEN* and *PDCD4* are the main targets of miR-21, which directly regulates them at the post-transcriptional level by binding to their 3′-UTRs. This interaction recruits the RNA-induced silencing complex (RISC), resulting in either mRNA degradation or translational repression [55]. *PTEN* is a well-known tumor suppressor gene involved in the regulation of the PI3K/AKT pathway, a key signal transduction pathway that controls cell growth and survival. *PTEN* dephosphorylates PIP3 to PIP2, inhibiting the downstream activation of AKT and mTOR signaling. Loss of *PTEN* activity due to miR-21 overexpression leads to increased PI3K/AKT pathway activity, which promotes oncogenic processes such as cell proliferation, survival, and metastasis. In addition, AKT is activated by miR-21 and partially inactivated by *PTEN* [2,44]. Similarly, *PDCD4* is another tumor suppressor gene that inhibits translation initiation and functions to suppress tumorigenesis by inhibiting cell growth and inducing apoptosis. Furthermore, *PDCD4* interacts with eukaryotic translation initiation factor 4A (eIF4A), a helicase required for cap-dependent mRNA translation, thereby suppressing the protein synthesis required for tumor growth, and which inhibits the expression of genes involved in matrix degradation, such as MMPs (matrix metalloproteinases). Therefore, reduced *PDCD4* expression correlates with increased invasion, metastasis, and a poor prognosis in cancer patients. We observed elevated expression levels of *PTEN* and *PDCD4* in miR-21 KO A549 cells. Li L et al. [56] revealed that up-regulation of miR-21 decreased the *PTEN* expression level, while negative regulation of miR-21 increased this in A549 cells, and they concluded that *PTEN* was a direct target of miR-21. Zheng et al. [57] used propofol, an inhibitor of miR-21, in non-small-cell lung cancer cells. Inhibition of miR-21 by propofol blocked the *PTEN*/AKT signaling pathway and increased the expression of *PTEN*, resulting in decreased cell growth and activation of apoptosis [58]. As for *PDCD4*, research was carried out in the PC9 NSCLC, showing that inhibiting miR-21 expression in these cells resulted in an increase in *PDCD4* expression [59]. In addition, Yang et al. [60] found an increase in *PDCD4* expression in A549 cells treated with an anti-miR-21 sponge compared to non-transfected A549 cells.

## 5. Conclusions

CRISPR/Cas9 technology can achieve high levels of miR-21 knock-out in A549 cells, thus reducing carcinogenic processes such as proliferation, migration, and colony formation. In addition, decreased miR-21 expression contributes to increased sensitivity of tumor cells to carboplatin and paclitaxel. Likewise, *PTEN* and *PDCD4*, two target genes of miR-21, increased in expression when this microRNA was inhibited. Therefore, miR-21 knock-out by CRISPR/Cas9 could be considered a potential strategy for future research into lung cancer treatment.

## Data Availability

The data presented in this study are available in the main article.

## References

[1] Yang I.V., Schwartz D.A. (2011). Epigenetic Control of Gene Expression in the Lung. Am. J. Respir. Crit. Care Med..

[2] Li H., Zhao J., Jia X., Zhang Y., Du Y., Li H., Ma L., Huang J. (2020). MiR-21 Promotes Growth, Invasion and Migration of Lung Cancer Cells by AKT/P-AKT/Cleaved-Caspase 3/MMP-2/MMP-9 Signaling Pathway. Int. J. Clin. Exp. Pathol..

[3] Sung H., Ferlay J., Siegel R.L., Laversanne M., Soerjomataram I., Jemal A., Bray F. (2021). Global Cancer Statistics 2020: GLOBOCAN Estimates of Incidence and Mortality Worldwide for 36 Cancers in 185 Countries. CA Cancer J. Clin..

[4] Pelosi G., Sonzogni A., Viale G. (2010). The Classification of Lung Carcinoma: Time to Change the Morphology-Based Approach?. Int. J. Surg. Pathol..

[5] Menon A., Abd-Aziz N., Khalid K., Poh C.L., Naidu R. (2022). MiRNA: A Promising Therapeutic Target in Cancer. Int. J. Mol. Sci..

[6] He B., Zhao Z., Cai Q., Zhang Y., Zhang P., Shi S., Xie H., Peng X., Yin W., Tao Y. (2020). MiRNA-Based Biomarkers, Therapies, and Resistance in Cancer. Int. J. Biol. Sci..

[7] Abdelaal A.M., Sohal I.S., Iyer S., Sudarshan K., Kothandaraman H., Lanman N.A., Low P.S., Kasinski A.L. (2023). A First-in-Class Fully Modified Version of MiR-34a with Outstanding Stability, Activity, and Anti-Tumor Efficacy. Oncogene.

[8] Abdelaal A.M., Sohal I.S., Iyer S.G., Sudarshan K., Orellana E.A., Ozcan K.E., dos Santos A.P., Low P.S., Kasinski A.L. (2024). Selective Targeting of Chemically Modified MiR-34a to Prostate Cancer Using a Small Molecule Ligand and an Endosomal Escape Agent. Mol. Ther. Nucleic Acids.

[9] Shi Y.P., Liu G.L., Li S., Liu X.L. (2020). MiR-17-5p Knockdown Inhibits Proliferation, Autophagy and Promotes Apoptosis in Thyroid Cancer via Targeting *PTEN*. Neoplasma.

[10] Pfeffer S.R., Yang C.H., Pfeffer L.M. (2015). The Role of MiR-21 in Cancer. Drug Dev. Res..

[11] Zhao W., Zhao J.J., Zhang L., Xu Q.F., Zhao Y.M., Shi X.Y., Xu A.G. (2015). Serum MiR-21 Level: A Potential Diagnostic and Prognostic Biomarker for Non-Small Cell Lung Cancer. Int. J. Clin. Exp. Med..

[12] Gao W., Lu X., Liu L., Xu J., Feng D., Shu Y. (2012). MiRNA-21: A Biomarker Predictive for Platinum-Based Adjuvant Chemotherapy Response in Patients with Non-Small Cell Lung Cancer. Cancer Biol. Ther..

[13] Ribas J., Ni X., Castanares M., Liu M.M., Esopi D., Yegnasubramanian S., Rodriguez R., Mendell J.T., Lupold S.E. (2012). A Novel Source for MiR-21 Expression through the Alternative Polyadenylation of VMP1 Gene Transcripts. Nucleic Acids Res..

[14] Zhou C., Wu L., Fan Y., Wang Z., Liu L., Chen G., Zhang L., Huang D., Cang S., Yang Z. (2021). Sintilimab Plus Platinum and Gemcitabine as First-Line Treatment for Advanced or Metastatic Squamous NSCLC: Results From a Randomized, Double-Blind, Phase 3 Trial (ORIENT-12). J. Thorac. Oncol..

[15] Vasconcellos V.F., Marta G.N., da Silva E.M., Gois A.F., de Castria T.B., Riera R. (2020). Cisplatin versus Carboplatin in Combination with Third-Generation Drugs for Advanced Non-Small Cell Lung Cancer. Cochrane Database Syst. Rev..

[16] Bi Y., Li F., Ren J., Han X. (2022). The Safety and Efficacy of Oxaliplatin-Loaded Drug-Eluting Beads Transarterial Chemoembolization for the Treatment of Unresectable or Advanced Lung Cancer. Front. Pharmacol..

[17] Shi M., Gu A., Tu H., Huang C., Wang H., Yu Z., Wang X., Cao L., Shu Y., Yang R. (2021). Comparing Nanoparticle Polymeric Micellar Paclitaxel and Solvent-Based Paclitaxel as First-Line Treatment of Advanced Non-Small-Cell Lung Cancer: An Open-Label, Randomized, Multicenter, Phase III Trial. Ann. Oncol..

[18] Min H.Y., Lee H.Y. (2021). Mechanisms of Resistance to Chemotherapy in Non-Small Cell Lung Cancer. Arch. Pharmacal Res..

[19] Sun L.H., Tian D., Yang Z.C., Li J.L. (2020). Exosomal MiR-21 Promotes Proliferation, Invasion and Therapy Resistance of Colon Adenocarcinoma Cells through Its Target *PDCD4*. Sci. Rep..

[20] Chen Z., Yuan Y.C., Wang Y., Liu Z., Chan H.J., Chen S. (2015). Down-Regulation of Programmed Cell Death 4 (*PDCD4*) Is Associated with Aromatase Inhibitor Resistance and a Poor Prognosis in Estrogen Receptor-Positive Breast Cancer. Breast Cancer Res. Treat..

[21] Wei Z.T., Zhang X., Wang X.Y., Gao F., Zhou C.J., Zhu F.L., Wang Q., Gao Q., Ma C.H., Sun W.S. (2009). *PDCD4* Inhibits the Malignant Phenotype of Ovarian Cancer Cells. Cancer Sci..

[22] De Marco C., Laudanna C., Rinaldo N., Oliveira D.M., Ravo M., Weisz A., Ceccarelli M., Caira E., Rizzuto A., Zoppoli P. (2017). Specific Gene Expression Signatures Induced by the Multiple Oncogenic Alterations That Occur within the *PTEN*/PI3K/AKT Pathway in Lung Cancer. PLoS ONE.

[23] Fu X., He Y., Wang X., Peng D., Chen X., Li X., Wang Q. (2017). Overexpression of MiR-21 in Stem Cells Improves Ovarian Structure and Function in Rats with Chemotherapy-Induced Ovarian Damage by Targeting *PDCD4* and *PTEN* to Inhibit Granulosa Cell Apoptosis. Stem Cell Res. Ther..

[24] Tirpe A., Gulei D., Razvan Tirpe G., Nutu A., Irimie A., Campomenosi P., Ancutapop L., Berindan-Neagoe I. (2020). Beyond Conventional: The New Horizon of Anti-Angiogenic MicroRNAs in Non-Small Cell Lung Cancer Therapy. Int. J. Mol. Sci..

[25] Toscano M.G., Anderson P., Muñoz P., Lucena G., Cobo M., Benabdellah K., Gregory P.D., Holmes M.C., Martin F. (2013). Use of Zinc-Finger Nucleases to Knock out the WAS Gene in K562 Cells: A Human Cellular Model for Wiskott-Aldrich Syndrome. DMM Dis. Models Mech..

[26] Toscano M.G., Muñoz P., Sánchez-Gilabert A., Cobo M., Benabdellah K., Anderson P., Ramos-Mejía V., Real P.J., Neth O., Molinos-Quintana A. (2016). Absence of WASp Enhances Hematopoietic and Megakaryocytic Differentiation in a Human Embryonic Stem Cell Model. Mol. Ther..

[27] Hsu P.D., Scott D.A., Weinstein J.A., Ran F.A., Konermann S., Agarwala V., Li Y., Fine E.J., Wu X., Shalem O. (2013). DNA Targeting Specificity of RNA-Guided Cas9 Nucleases. Nat. Biotechnol..

[28] Ran F.A., Hsu P.D., Wright J., Agarwala V., Scott D.A., Zhang F. (2013). Genome Engineering Using the CRISPR-Cas9 System. Nat. Protoc..

[29] Maldonado-Pérez N., Tristán-Manzano M., Justicia-Lirio P., Martínez-Planes E., Muñoz P., Pavlovic K., Cortijo-Gutiérrez M., Blanco-Benítez C., Castella M., Juan M. (2022). Efficacy and Safety of Universal (TCRKO) ARI-0001 CAR-T Cells for the Treatment of B-Cell Lymphoma. Front. Immunol..

[30] Gutierrez-Guerrero A., Sanchez-Hernandez S., Galvani G., Pinedo-Gomez J., Martin-Guerra R., Sanchez-Gilabert A., Aguilar-González A., Cobo M., Gregory P., Holmes M. (2018). Comparison of Zinc Finger Nucleases Versus CRISPR-Specific Nucleases for Genome Editing of the Wiskott-Aldrich Syndrome Locus. Hum. Gene Ther..

[31] Sánchez-Hernández S., Aguilar-González A., Guijarro-Albaladejo B., Maldonado-Pérez N., Ramos-Hernández I., Cortijo-Gutiérrez M., Sánchez Martín R.M., Benabdellah K., Martin F. (2020). Development of Cellular Models to Study Efficiency and Safety of Gene Edition by Homologous Directed Recombination Using the CRISPR/Cas9 System. Cells.

[32] Benabdellah K., Sánchez-Hernández S., Aguilar-González A., Maldonado-Pérez N., Gutierrez-Guerrero A., Cortijo-Gutierrez M., Ramos-Hernández I., Tristán-Manzano M., Galindo-Moreno P., Herrera C. (2020). Genome-Edited Adult Stem Cells: Next-Generation Advanced Therapy Medicinal Products. Stem Cells Transl. Med..

[33] Sánchez-Rivera F.J., Jacks T. (2015). Applications of the CRISPR–Cas9 System in Cancer Biology. Nat. Rev. Cancer.

[34] Tzelepis K., Koike-Yusa H., De Braekeleer E., Li Y., Metzakopian E., Dovey O.M., Mupo A., Grinkevich V., Li M., Mazan M. (2016). A CRISPR Dropout Screen Identifies Genetic Vulnerabilities and Therapeutic Targets in Acute Myeloid Leukemia. Cell Rep..

[35] Sorrentino C., D’Antonio L., Ciummo S.L., Fieni C., Landuzzi L., Ruzzi F., Vespa S., Lanuti P., Lotti L.V., Lollini P.L. (2022). CRISPR/Cas9-Mediated Deletion of Interleukin-30 Suppresses IGF1 and CXCL5 and Boosts SOCS3 Reducing Prostate Cancer Growth and Mortality. J. Hematol. Oncol..

[36] D’Antonio L., Fieni C., Ciummo S.L., Vespa S., Lotti L., Sorrentino C., Di Carlo E. (2023). Inactivation of Interleukin-30 in Colon Cancer Stem Cells via CRISPR/Cas9 Genome Editing Inhibits Their Oncogenicity and Improves Host Survival. J. Immunother. Cancer.

[37] McAndrews K.M., Xiao F., Chronopoulos A., LeBleu V.S., Kugeratski F.G., Kalluri R. (2021). Exosome-Mediated Delivery of CRISPR/Cas9 for Targeting of Oncogenic KrasG12D in Pancreatic Cancer. Life Sci. Alliance.

[38] Soucek L., Whitfield J., Martins C.P., Finch A.J., Murphy D.J., Sodir N.M., Karnezis A.N., Swigart L.B., Nasi S., Evan G.I. (2008). Modelling Myc Inhibition as a Cancer Therapy. Nature.

[39] Menegatti J., Nakel J., Stepanov Y.K., Caban K.M., Ludwig N., Nord R., Pfitzner T., Yazdani M., Vilimova M., Kehl T. (2022). Changes of Protein Expression after CRISPR/Cas9 Knockout of MiRNA-142 in Cell Lines Derived from Diffuse Large B-Cell Lymphoma. Cancers.

[40] Lin S.C., Wu H.L., Yeh L.Y., Yang C.C., Kao S.Y., Chang K.W. (2020). Activation of the MiR-371/372/373 MiRNA Cluster Enhances Oncogenicity and Drug Resistance in Oral Carcinoma Cells. Int. J. Mol. Sci..

[41] Caramori G., Casolari P., Cavallesco G.N., Giuffr S., Adcock I., Papi A. (2011). Mechanisms Involved in Lung Cancer Development in COPD. Int. J. Biochem. Cell Biol..

[42] Volinia S., Calin G.A., Liu C.G., Ambs S., Cimmino A., Petrocca F., Visone R., Iorio M., Roldo C., Ferracin M. (2006). A MicroRNA Expression Signature of Human Solid Tumors Defines Cancer Gene Targets. Proc. Natl. Acad. Sci. USA.

[43] Li J., Huang H., Sun L., Yang M., Pan C., Chen W., Wu D., Lin Z., Zeng C., Yao Y. (2009). MiR-21 Indicates Poor Prognosis in Tongue Squamous Cell Carcinomas as an Apoptosis Inhibitor. Clin. Cancer Res..

[44] Dai L., Chen F., Zheng Y., Zhang D., Qian B., Ji H., Long F., Cretoiu D. (2019). MiR-21 Regulates Growth and EMT in Lung Cancer Cells via *PTEN*/Akt/GSK3β Signaling. Front. Biosci..

[45] Rama A.R., Quiñonero F., Mesas C., Melguizo C., Prados J. (2022). Synthetic Circular MiR-21 Sponge as Tool for Lung Cancer Treatment. Int. J. Mol. Sci..

[46] Li S., Zeng X., Ma R., Wang L. (2018). MicroRNA-21 Promotes the Proliferation, Migration and Invasion of Non-Small Cell Lung Cancer A549 Cells by Regulating Autophagy Activity via AMPK/ULK1 Signaling Pathway. Exp. Ther. Med..

[47] Huo W., Zhao G., Yin J., Ouyang X., Wang Y., Yang C., Wang B., Dong P., Wang Z., Watari H. (2017). Lentiviral CRISPR/Cas9 Vector Mediated MiR-21 Gene Editing Inhibits the Epithelial to Mesenchymal Transition in Ovarian Cancer Cells. J. Cancer.

[48] Nieland L., van Solinge T.S., Cheah P.S., Morsett L.M., El Khoury J., Rissman J.I., Kleinstiver B.P., Broekman M.L.D., Breakefield X.O., Abels E.R. (2022). CRISPR-Cas Knockout of MiR21 Reduces Glioma Growth. Mol. Ther. Oncolytics.

[49] Liu H., Liu T., Zhou Y., Song X., Wei R. (2020). Overexpression of Long Non-Coding RNA Cancer Susceptibility 11 Is Involved in the Development of Chemoresistance to Carboplatin in Hepatocellular Carcinoma. Oncol. Lett..

[50] Alharbi M., Sharma S., Guanzon D., Lai A., Zuñiga F., Shiddiky M.J.A., Yamauchi Y., Salas-Burgos A., He Y., Pejovic T. (2020). MiRNa Signature in Small Extracellular Vesicles and Their Association with Platinum Resistance and Cancer Recurrence in Ovarian Cancer. Nanomedicine.

[51] Gamal-Eldeen A.M., Alrehaili A.A., Alharthi A., Raafat B.M. (2022). Perftoran Inhibits Hypoxia-Associated Resistance in Lung Cancer Cells to Carboplatin. Front. Pharmacol..

[52] Su C., Cheng X., Li Y., Han Y., Song X., Yu D., Cao X., Liu Z. (2018). MiR-21 Improves Invasion and Migration of Drug-resistant Lung Adenocarcinoma Cancer Cell and Transformation of EMT through Targeting HBP1. Cancer Med..

[53] Du G., Cao D., Meng L. (2017). MiR-21 Inhibitor Suppresses Cell Proliferation and Colony Formation through Regulating the *PTEN*/AKT Pathway and Improves Paclitaxel Sensitivity in Cervical Cancer Cells. Mol. Med. Rep..

[54] Tao L., Wu Y.Q., Zhang S.P. (2019). MiR-21-5p Enhances the Progression and Paclitaxel Resistance in Drug-Resistant Breast Cancer Cell Lines by Targeting *PDCD4*. Neoplasma.

[55] Farasati Far B., Vakili K., Fathi M., Yaghoobpoor S., Bhia M., Naimi- Jamal M.R. (2023). The Role of MicroRNA-21 (MiR-21) in Pathogenesis, Diagnosis, and Prognosis of Gastrointestinal Cancers: A Review. Life Sci..

[56] Li L., Zhang H., Wang X., Wang J., Wei H. (2018). Long Non-Coding RNA CASC2 Enhanced Cisplatin-Induced Viability Inhibition of Non-Small Cell Lung Cancer Cells by Regulating the *PTEN*/PI3K/Akt Pathway through down-Regulation of MiR-18a and MiR-21. RSC Adv..

[57] Zheng X., Dong L., Zhao S., Li Q., Liu D., Zhu X., Ge X., Li R., Wang G. (2020). Propofol Affects Non–Small-Cell Lung Cancer Cell Biology By Regulating the MiR-21/*PTEN*/AKT Pathway In Vitro and In Vivo. Anesth. Analg..

[58] Ding Y., Hou Y., Liu Y., Xie X., Cui Y., Nie H. (2021). Prospects for MiR-21 as a Target in the Treatment of Lung Diseases. Curr. Pharm. Des..

[59] Li B., Ren S., Li X., Wang Y., Garfield D., Zhou S., Chen X., Su C., Chen M., Kuang P. (2014). MiR-21 Overexpression Is Associated with Acquired Resistance of EGFR-TKI in Non-Small Cell Lung Cancer. Lung Cancer.

[60] Yang Y., Meng H., Peng Q., Yang X., Gan R., Zhao L., Chen Z., Lu J., Meng Q.H. (2014). Downregulation of MicroRNA-21 Expression Restrains Non-Small Cell Lung Cancer Cell Proliferation and Migration through Upregulation of Programmed Cell Death 4. Cancer Gene Therapy.

